# Coronavirus Resistance Database (CoV-RDB): SARS-CoV-2 susceptibility to monoclonal antibodies, convalescent plasma, and plasma from vaccinated persons

**DOI:** 10.1371/journal.pone.0261045

**Published:** 2022-03-09

**Authors:** Philip L. Tzou, Kaiming Tao, Sergei L. Kosakovsky Pond, Robert W. Shafer

**Affiliations:** 1 Division of Infectious Diseases, Stanford University School of Medicine, Stanford, CA, United States of America; 2 Institute for Genomics and Evolutionary Medicine, Temple University, Philadelphia, PA, United States of America; Translational Health Science & Technology Institute, INDIA

## Abstract

As novel SARS-CoV-2 variants with different patterns of spike protein mutations have emerged, the susceptibility of these variants to neutralization by antibodies has been rapidly assessed. However, neutralization data are generated using different approaches and are scattered across different publications making it difficult for these data to be located and synthesized. The Stanford Coronavirus Resistance Database (CoV-RDB; https://covdb.stanford.edu) is designed to house comprehensively curated published data on the neutralizing susceptibility of SARS-CoV-2 variants and spike mutations to monoclonal antibodies (mAbs), convalescent plasma (CP), and vaccinee plasma (VP). As of December 31, 2021, CoV-RDB encompassed 257 publications including 91 (35%) containing 9,070 neutralizing mAb susceptibility results, 131 (51%) containing 16,773 neutralizing CP susceptibility results, and 178 (69%) containing 33,540 neutralizing VP results. The database also records which spike mutations are selected during *in vitro* passage of SARS-CoV-2 in the presence of mAbs and which emerge in persons receiving mAbs as treatment. The CoV-RDB interface interactively displays neutralizing susceptibility data at different levels of granularity by filtering and/or aggregating query results according to one or more experimental conditions. The CoV-RDB website provides a companion sequence analysis program that outputs information about mutations present in a submitted sequence and that also assists users in determining the appropriate mutation-detection thresholds for identifying non-consensus amino acids. The most recent data underlying the CoV-RDB can be downloaded in its entirety from a GitHub repository in a documented machine-readable format.

## Introduction

Beginning in late 2020, several SARS-CoV-2 variants sharing multiple spike mutations were reported from different parts of the world. These variants have been classified according to their phylogenetic lineage and component mutations. Variants that spread widely and displayed evidence for being more transmissible, causing more severe disease and/or reducing neutralization by antibodies generated during previous infection or vaccination have been classified as variants of concern (VOCs) by the World Health Organization and U.S. Centers for Disease Control and Prevention (reviewed in [1]). Variants that have spread less widely but share some of the key mutations present within VOCs have been classified as variants of interest (VOIs).

As novel SARS-CoV-2 variants have emerged, numerous investigations assessed the susceptibility of individual and combination spike mutations to neutralization by monoclonal antibodies (mAbs), convalescent plasma (CP), and vaccinee plasma (VP). The susceptibility of SARS-CoV-2 variants to mAbs is obviously relevant for preventing and treating SARS-CoV-2 infections with mAb regimens. The susceptibility of SARS-CoV-2 variants to CP provides insight into the likelihood that a variant will infect and cause illness in a person previously infected with and recovered from a different variant. The susceptibility of SARS-CoV-2 variants to VP provides insight into the risk that a variant will infect and cause illness in a previously vaccinated person. Ultimately, however, the risks of re-infection and vaccine breakthrough, and the nature of the ensuing illness must be assessed in epidemiological studies.

The SARS-CoV-2 spike protein is a 1,273-amino acid trimeric glycoprotein responsible for entry into host cells. Each spike monomer has a largely exposed S1 attachment domain (residues 1–686) and a partially buried S2 fusion domain (residues 687–1,273) [2, 3]. Part of S1, called the receptor-binding domain (RBD; residues 306–534) binds to the human angiotensin-converting enzyme 2 (ACE2) receptor [4, 5]. Approximately 20 RBD residues bind the ACE2 receptor. The part of the RBD containing these residues (438–506) is referred to as the receptor-binding motif (RBM), whereas the remainder of the RBD is called the RBD core. The SARS-CoV-2 RBD is the main target of neutralizing antibodies [6, 7]. Like the RBD, much of the S1 amino-terminal domain (NTD) is also exposed on the spike trimer surface and is targeted by neutralizing antibodies.

In April 2020, we created a relational database that we called the Stanford Coronavirus Antiviral Research Database containing *in vitro*, animal model, and clinical trial data intended to promote uniform reporting of experimental results, to facilitate comparisons between candidate antiviral compounds, and to objectively synthesize published antiviral research [8]. With the emergence of SARS-CoV-2 variants having new biological and epidemiological characteristics, we created a second database containing curated neutralizing susceptibility data of SARS-CoV-2 mutations and variants to mAbs, CP, and VP. The website was renamed the Stanford Coronavirus Antiviral & Resistance Database but maintained the same acronym (CoV-RDB). The database includes specific data on which spike mutations have been selected during both i*n vitro* passage of SARS-CoV-2 in the presence of mAbs and *in vivo* emergence in persons receiving mAbs for treatment. The CoV-RDB website enables users to query its database and the associated GitHub repository enables users to download the entire database. The CoV-RDB website also contains a companion sequence analysis program that annotates SARS-CoV-2 user-submitted sequences using CoV-RDB data.

## Methods and results

The CoV-RDB data model is made up of four major entities: published references, viral mutations and variants, antibodies (including mAbs, CP, and VP), and experimental results. As of December 2021, the database contains 257 publications: 19 (7%) with 427 results from *in vitro* mAb selection experiments, 91 (35%) with 9,070 neutralizing mAb susceptibility results, 131 (51%) containing 16,773 with CP susceptibility results, and178 (69%) with 33,540 neutralizing VP results. As publications often contain more than one type of experiment, the sum of the percentages is greater than 100%. The complete database schema is available at https://github.com/hivdb/covid-drdb/blob/master/schema.dbml.

### Curation of published references

Published references included in the CoV-RDB are obtained weekly from three literature sources: i) PubMed using the search term “SARS-CoV-2”; ii) BioRxiv/MedRxiv COVID-19 SARS-CoV-2 preprint servers; and iii) the Research Square SARS-CoV-2/COVID-19 preprint server. Publication titles and abstracts are reviewed manually to identify studies containing data on SARS-CoV-2 spike mutations and humoral immunity. Studies that pass initial review are downloaded to a Zotero reference database and full texts are reviewed manually to extract specific data on the selection of spike mutations *in vitro* and *in vivo*, and neutralizing susceptibility data for individual spike mutations or combinations of spike mutations, such as those present in SARS-CoV-2 variants to mAbs, CP, and VP. As of December 31, 2021, 57 (22.2%) of 257 references were preprints including 13 (5.1%) published online during the first six months of 2021 and 44 (17.1%) during the last six months of 2021. Studies initially published as preprints are re-reviewed following peer-review publication. Studies that remain unpublished for more than one year will be evaluated for continued inclusion in the database.

For each publication, data are first entered into linked comma-separated files (CSVs) contained in a Github repository (https://github.com/hivdb/covid-drdb-payload). Data are then imported into a PostgreSQL database where they are validated for completeness and consistency. The data in the PostgreSQL database are then exported as a single SQLite database file that is both available for download under the CC BY-SA 4.0 open-source license and used as the source for regularly updated datasets and queries on the CoV-RDB website. The entire workflow is summarized in S1 Fig.

### Virus variants and mutations

In the CoV-RDB data model, the virus entity represents individual spike mutations, combinations of spike mutations, and virus variants for which the full set of spike and genomic mutations is known including VOCs and VOIs. Published *in vitro* neutralization experiments have been performed using (i) replication-competent primary SARS-CoV-2 isolates [9]; (ii) replication-competent full-length cloned recombinant SARS-CoV-2 viruses generated using multiple plasmids, or bacterial or yeast artificial chromosomes [10]; (iii) replication-competent chimeric SARS-CoV-2 viruses in a vesicular stomatitis virus (VSV) genomic backbone containing a spike protein with specific mutations [11]; (iv) non-replication-competent pseudotyped viruses using VSV or a lentivirus containing a SARS-CoV-2 spike protein with specific mutations [12–14]; and (v) surrogate neutralizing assays based on antibody-mediated blocking of the RBD-ACE2 interaction [15–17].

For primary SARS-CoV-2 isolates, the virus is also characterized by mutations in other viral proteins that may influence viral replication kinetics but not neutralization susceptibility. For pseudotyped, chimeric, and recombinant viruses, the virus can be characterized entirely by its spike mutations. For surrogate neutralization assays, the virus is generally characterized only by the virus’s RBD mutations. Most *in vitro* selection experiments have been performed using chimeric VSVs containing SARS-CoV-2 spike proteins as these viral constructs can undergo multiple rounds of replication in cell culture.

Table 1 lists the VOCs, VOIs, and other full-genomic SARS-CoV-2 variants for which data are available in CoV-RDB. Approximately 83% of results on variants are for VOCs, 11% are for VOIs, and 6% are for other variants containing one or more RBD mutations. As SARS-CoV-2 variants are continually evolving and variant definitions are regularly updated, individual VOCs and VOIs often differ slightly in the mutations that they contain. S1 Table displays the variability in these patterns for each of the VOCs and VOIs. For example, the alpha, beta, gamma, delta, and omicron variants in CoV-RDB contain 15, 28, 8, 22, and 7 distinct patterns of spike mutations, respectively, all closely related to an archetypal consensus variant.

**Table 1:** Variants of concern, variants of interest, and other variants for which neutralization data are available in CoV-RDB.

Virus types	References	Results
***Variants of concern***		
Alpha (B.1.1.7)	140	9505
Beta (B.1.351)	155	10933
Gamma (P.1)	88	5015
Delta (B.1.617.2)	82	4971
Omicron (B.1.1.529)	31	2855
***Variants of interest***		
Epsilon (B.1.427/B.1.429)	29	1323
Eta (B.1.525)	11	238
Iota (B.1.526)	22	1099
Kappa (B.1.617.1)	32	1223
Lambda (C.37)	17	508
Mu (B.1.621)	10	171
***Other variants containing ≥1 RBD mutation (n = 17)***		
B.1.463 [F384L]	1	220
B.1.1.298 [Y453F]	7	215
B.1.617.3 [L452R, E484Q]	3	106
B.1.1.241 [S477N]	1	64
B.1/E484K	1	32
R.1 [E484K]	3	271
B.1.1.141 [T385I]	1	17
B.1.1.317 [S477N, A522S]	1	14
P.2 [E484K]	7	293
B.1.1.318 [E484K]	1	45
B.1.618 [E484K]	1	20
A.23.1 [V367F, E484K]	3	27
B.1.1.519 [T478K]	3	32
A.30 [R346K, T478R, E484K]	3	55
A.27/A222V [L452R, N501Y]	1	6
B.1.619 [N440K, E484K]	1	1
R.2 [E484K]	1	1

The most common ancestral variants lacking immune escape mutations used as controls include lineage A variants (n = 4,440), lineage B variants (Wuhan consensus; n = 27,045), lineage B.1 variants (Wuhan consensus + D614G; n = 25,213).

Table 2 lists the most common studied individual mutations within the spike RBD, NTD, C-terminal domain (CTD), and S2 domain. These mutations have generally been studied one at a time within an ancestral virus backbone. CoV-RDB contains experimental data on 540 different individual spike mutations at 204 positions including 373 RBD mutations at 94 positions, 96 NTD mutations at 57 positions, 55 S2 mutations at 40 positions, and 16 CTD mutations at 13 positions. CoV-RDB also contains results on 206 combinations of spike mutations in addition to VOCs and VOIs. These generally consist of sets of two or three mutations that are present within a VOC or VOI. Several of the most common mutation combinations are listed in the footnote of Table 2.

**Table 2:** Individual SARS-CoV-2 spike mutations for which data are available in CoV-RDB.

Mutations	References	Results
***Receptor-binding domain (RBD)***		
E484K	45	832
N501Y	37	954
K417N	27	667
L452R	22	383
N439K	21	299
S477N	21	340
Y453F	17	291
F490S	15	149
E484Q	15	224
G446V	15	147
A475V	12	186
N440K	9	192
T478K	9	128
Other mutations	43	6658
***N-terminal domain (NTD)***		
Δ69/70	13	308
Δ242	6	172
L18F	7	136
D215G	4	126
R246I	4	116
D80A	3	114
Δ144	4	113
Other mutations	23	856
***C-terminal domain of S1 (CTD)***		
P681H	8	130
A570D	4	88
P681R	2	29
H655Y	3	32
Other mutations	6	148
***S2***		
A701V	3	113
S982A	4	93
D1118H	4	88
T716I	4	87
D936Y	3	60
Other mutations	14	688

The most commonly studied mutation combinations that are not VOCs or VOIs included K417N+E484K+N501Y (n = 25 studies; 975 results), E484K+N501Y (n = 5 studies; 189 results), Δ69/70+N501Y (n = 8 studies; 158 results), Δ69/70+Y453F (n = 3 studies; 77 results), L452R+E484Q+P681R (n = 3 studies; 55 results) K417N+N501Y (n = 2 studies; 75 results), K417N+E484K (n = 2 studies; 75 results), and L452R+E484Q (n = 8 studies; 129 results).

### Antibodies (mAbs, CP, and VP)

#### mAbs

**Fig 1:**
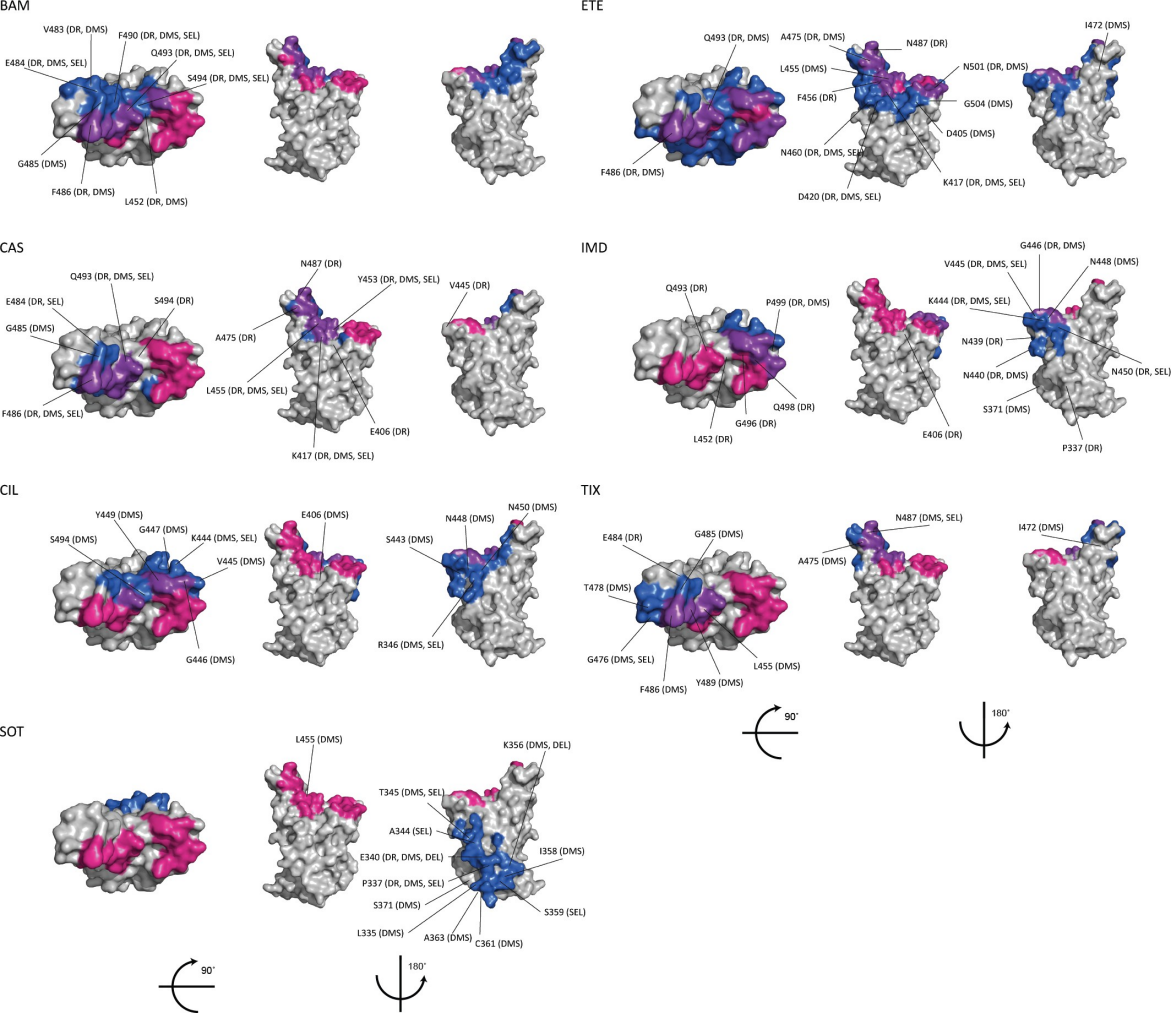
Monoclonal Antibodies (mAbs) with EUAs or in advanced clinical development: Receptor Binding Domain (RBD) epitopes and immune escape positions. For each mAb, the top of the RBD and two side views are depicted using coordinates from PDB 6M0J. ACE2 binding residues are shown in red; the mAb epitope defined as those residues within 4.5 angstroms of the mAb paratope is shown in dark blue; and ACE2 binding residues within the mAb epitope are shown in purple. Those positions containing mutations that were either selected by the mAb *in vitro* (“SEL”), reduced binding in a deep mutational scanning assay (“DMS”), and/or reduced *in vitro* neutralizing susceptibility by a median of ≥4-fold in CoV-RDB (drug resistance; “DR”) are also indicated. The mAb epitopes for BAM (bamlanivimab), ETE (etesevimab), CAS (casirivimab), IMD (imdevimab), SOT (sotrovirmab), CIL (cilgavimab) and TIX (tixagevimab) were determined from their PDB structures.

In CoV-RDB, mAbs are characterized according to their stage of clinical development; spike target; and specific epitope, i.e., the list of amino acids within 4.5 angstroms of the mAb paratope, according to structural data obtained from the Protein DataBank (PDB). Table 3 lists those mAbs with FDA emergency use authorizations (EUAs) or that have been approved in one or more countries along with their spike targets and numbers of experimental results including *in vitro* selection and neutralization susceptibility data [18]. CoV-RDB contains 2,526 results for the ten mAbs in Table 3, 352 results for six additional investigational mAbs in clinical trials at the time of writing, and 6,512 results for 373 mAbs that are not in clinical development as of December 2021 [19]. For 64 of these 373 additional mAbs, structural data are available in the PDB. Table 4 summarizes neutralization susceptibilities for the seven most studied variants and the six most studied individual spike mutations to those mAbs that either have EUAs or that have been approved in one or more countries. Fig 1 shows the relationship between mAb epitopes, mutations selected during *in vitro* passage, and mutations that reduce mAb binding and/or susceptibility.

**Table 3:** Monoclonal Antibodies (mAbs) with Emergency Use Authorization (EUA) or approved for use outside of the U.S.

mAb	Company	Stage	PDB	Target^1^	Publication^2^	Results^2^
Bamlanivimab	Lilly	EUA	7KMG	RBM II	31	220
Etesevimab	Lilly	EUA	7C01	RBM I	33	356
Casirivimab	Regeneron	EUA	6XDG	RBM I	45	408
Imdevimab	Regeneron	EUA	6XDG	RBD core	45	407
Sotrovimab	Vir	EUA	6WPS	RBD core	27	345
Tixagevimab	AstraZeneca	EUA	7L7D	RBM I	20	107
Cilgavimab	AstraZeneca	EUA	7L7E	RBM II	21	126
BRII-196	BRII Biosciences	China	7CDI	RBM I	7	198
BRII-198	BRII Biosciences	China	N/A	RBD core	4	94
Regdanivimab	Celltrion	South Korea	7CM4	RBM I	9	30

^1^RBM (receptor binding motif) is the part of the receptor binding domain (RBD) that contains the spike ACE2 binding residues. RBM class 1 mAbs bind epitopes dominated by ACE2-binding residues and as a result bind solely when the RBD is in the up/open position. RBM class 2 mAbs have a smaller ACE2-binding footprint and can often bind the RBD in down/closed position. RBD core mAbs target a surface accessible part of the RBD that is separate from the RBM. ^2^The publications and results include those describing both *in vitro* selection and neutralization experiments.

**Table 4:** Median fold reduced neutralization susceptibility of 6 variants and 6 spike mutations to 12 mAbs in advanced clinical development^1^.

	BAM	ETE	CAS	IMD	CIL	TIX	SOT	REG	BRII-196	BRII-198
Alpha	1_13_	16_11_	1_19_	0.7_19_	1_8_	1.7_7_	2.3_15_	2.6_2_	0.6_4_	0.2_3_
Beta	>1000_15_	313_13_	76_23_	0.6_22_	1.1_7_	6.3_7_	1_14_	33_3_	0.6_6_	6_3_
Gamma	>1000_11_	294_11_	200_17_	0.4_16_	0.5_7_	6.4_6_	1.3_12_	61_3_	0.6_2_	0.7
Delta	>1000_12_	0.5_12_	0.7_13_	1.5_13_	3.5_4_	0.8_4_	1.3_8_	9.8_3_	0.8	-
Omicron	>1000_12_	>250_11_	>1000_13_	>500_13_	336_12_	735_12_	4.9_12_	>1000_5_	>140_3_	14_2_
Iota	>1000_5_	1.4_5_	11_4_	1.2_4_	0.9_2_	8.1_2_	0.8_4_	-	-	-
Epsilon	>1000_4_	1_4_	1.3_2_	1.7_2_	3	-	0.7_3_	43_4_	-	-
N501Y	1.1_5_	3.1_8_	1_9_	0.8_9_	1.1_5_	1.3_4_	1.7_8_	5.5	1_5_	2.3_4_
E484K	>1000_4_	2.9_7_	13_13_	1_13_	1.5_4_	4.6_4_	0.4_7_	8.7	1.4_4_	2.5_3_
K417N	0.5_4_	761_7_	7_9_	0.7_9_	0.6_5_	0.4_4_	0.6_7_	-	1.8_5_	0.5_4_
L452R	>1000_2_	1_5_	1_5_	2_6_	-	-	0.6	35	1.2_2_	200
F490S	293_2_	1.1_2_	0.8_3_	1.2_3_	-	-	0.8_2_	-	1.3	135
S494P	86_2_	0.6_2_	3.5_4_	1.2_3_	-	-	2_2_	-	0.7	1.6

^1^The subscript indicates the number of results from which the median fold-reduction in susceptibility was determined. Abbreviations: Bamlanivimab (BAM); Etesevimab (ETE); Casirivimab (CAS); Imdevimab (IMD); sotrovimab (SOT); Cilgavimab (CIL); Tixagevimab (TIX); Regdanivimab (REG).

#### Convalescent Plasma (CP)

In CoV-RDB, CP are characterized by the sequence of the infecting variant, severity of illness, and time since infection. Table 5 lists the numbers of experimental results in CoV-RDB according to these characteristics and the SARS-CoV-2 variant or mutation(s) tested for neutralization. Overall, 16,773 neutralization experiments were performed using CP samples (131 studies) including 113 studies that provided data for individual samples and 20 studies that provided only aggregate data.

**Table 5:** Numbers of Convalescent Plasma (CP) neutralization experiments in CoV-RDB according to the infecting virus, time since infection, and SARS-CoV-2 variant tested.

Characteristic	References	Results
***Infecting virus***		
Ancestral (A, B, B.1, others)	144	12213
Alpha	14	1303
Beta	14	463
Gamma	4	168
Delta	8	345
***Time since infection***		
1 month	105	12011
2–3 months	46	3322
4–6 months	26	1513
>6 months	18	1055
***Variant / mutations undergoing neutralization***		
Alpha	69	3178
Beta	77	3412
Gamma	36	1533
Delta	33	1022
Omicron	17	553
VOIs (kappa, iota, epsilon, lambda)	12	227
Other variants	27	1205
Individual mutations and mutation combinations	56	6003

The time since infection was available for all CP sample results including 16,846 samples obtained within six months of infection and 1,055 samples obtained beyond six months. The severity of illness was described for 4,613 (27%) CP samples with 2,754 samples from persons with mild-to-moderate disease and 1,859 samples from persons with severe disease. Although the sequence of the infecting virus was rarely known, the vast majority were obtained prior to the emergence of VOCs and VOIs. Nonetheless, 8%, 3%, 1%, and 2% were obtained from persons known to be infected with the Alpha, Beta, Gamma, and Delta variants, respectively.

#### Vaccine Plasma (VP)

In CoV-RDB, VP are characterized according to the vaccine received, number of vaccinations, time since vaccination, and whether the VP was obtained from a person who was also previously infected with SARS-CoV-2. Table 6 lists the numbers of experimental results according to each of the above characteristics and the variant or mutation(s) tested for neutralization. 65% of VP were obtained from persons receiving one of the two widely used mRNA vaccines (BNT162b2 and mRNA-1273) while about 15% were obtained from persons receiving the Coronovac, AZD1222, or Ad26.COV2.S vaccines. Approximately 81%, 17%, and 2% of VP samples were obtained within 1 month, 2–6 months, and >6 months after vaccination, respectively. Approximately 5% of samples were obtained from persons with confirmed infection prior to vaccination.

**Table 6:** Numbers of Vaccinee Plasma (VP) neutralization experiments in CoV-RDB according to the vaccine, history of previous infection, time since infection, and SARS-CoV-2 variant tested.

Characteristic	References	Results
***Vaccine***		
BNT162b2	121	16374
mRNA-1273	45	5569
CoronaVac	9	1626
AZD1222	26	2345
Ad26.COV2.S	13	1092
BBV152	6	569
Sputnik V	5	428
AZD1222 + BNT162b2	8	544
MVC-COV1901	1	342
BBV154	1	120
BBIBP-CorV	6	586
NVX-CoV2373	2	148
AZD1222 + AZD2816	1	59
ZF2001	2	51
mRNA-1273 + mRNA-1273.351	2	277
AZD2816	1	20
***Previously SARS-CoV-2 infected***		
Yes	34	1655
No	176	31885
***Time since vaccination***		
1 month	157	27310
2–3 months	35	3951
4–6 months	23	1746
> 6 months	16	533
***Variant / mutations undergoing neutralization***		
Alpha	92	5848
Beta	106	7150
Gamma	58	3247
Delta	63	3764
VOIs (kappa, iota, epsilon, lambda)	30	1484
Other variants	55	3496
Individual mutations and mutation combinations	78	7269
***Number of vaccinations***		
1	47	6742
2	163	24506
3	26	2292

The numbers of references and results obtained from persons receiving uncommonly studied vaccines or vaccine combinations are not shown.

**Fig 2:**
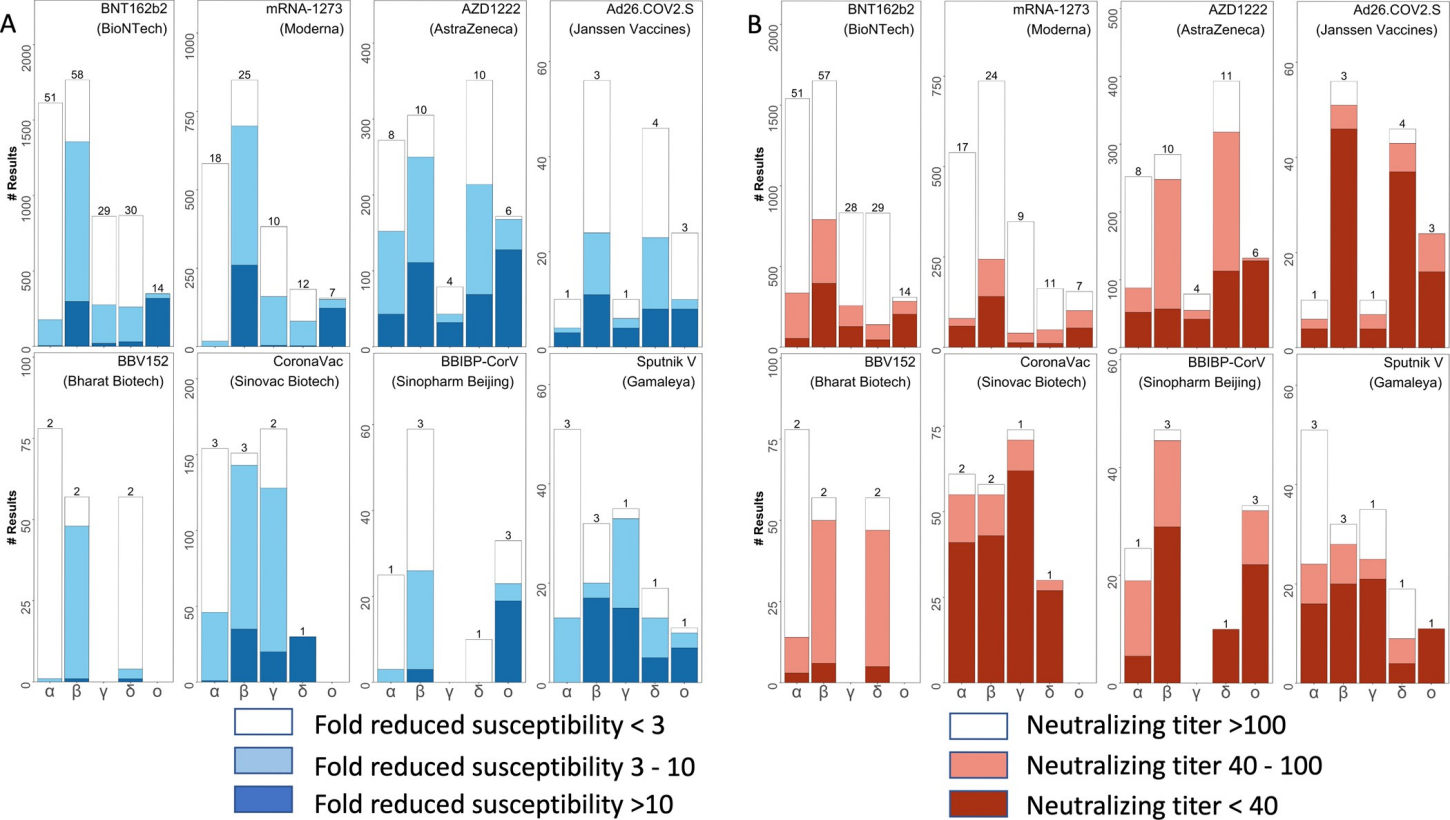
Distribution of fold-reduction in susceptibilities (A) and of absolute neutralizing titers (B) of vaccinee plasma (VP) associated with eight vaccines to the five variants of concern (VOC): Alpha, Beta, Gamma, Delta, and Omicron. The X axes indicate the Greek letter associated with each VOC. The Y axes indicate the number of neutralizing assays. The numbers above the stacked bars indicate the number of studies reporting the experimental results. The figure includes VP obtained solely from previously uninfected persons one month after receiving completing initial vaccination.

Fig 2 shows the distribution of fold-reductions in neutralizing susceptibilities and absolute neutralizing antibody titers against each of the VOCs for previously uninfected persons one month after completing the recommended course of vaccination for eight vaccines. Across all vaccines, each of the VOCs was found to have significantly different median neutralization titers (Kruskal-Wallis test; p<10^−6^ for all comparisons). A similar fold-reduction in neutralizing susceptibility against a given VOC is likely to be more consequential for those vaccines that elicit lower titers of neutralizing antibodies.

### CoV-RDB website

The CoV-RDB website contains four main features: (i) searchable tables containing SARS-CoV-2 variants, mAbs, vaccines, and references; (ii) regularly updated data summaries such as the data shown in Table 4 for mAbs and in Fig 2 for VP; (iii) user-defined queries; and (iv) a sequence analysis program. The user-defined queries and sequence analysis program are described here because they are interactive and contain multiple user options.

### User-defined queries

#### Query interface

**Fig 3:**
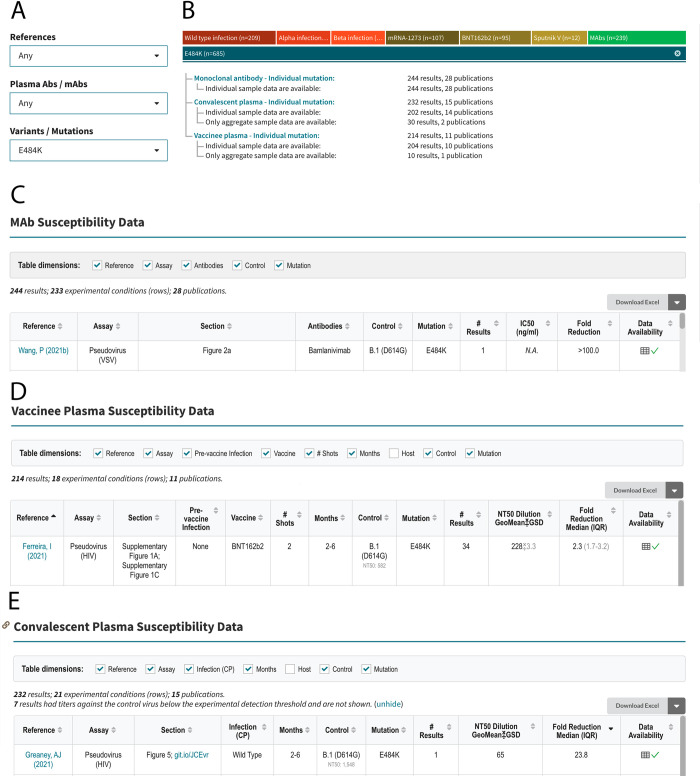
Query interface, sample query, and outline of query results for an example query for the SARS-CoV-2 RBD mutation E484K. The query interface containing three dropdown boxes is shown at the upper left (A). E484K is selected from the “Variants / Mutations” dropdown box. The upper right summarizes the data returned by the query, which in this case includes 244 mAb neutralizing susceptibility results from 28 publications, 232 convalescent plasma (CP) results from 15 publications, and 214 vaccinee plasma (VP) results from 11 publications (B). The summary distinguishes between results for which only aggregate (mean or median) data are provided and results for which individual data are provided. The sections below show the headers and first row of the tables containing mAb (C), VP (D), and CP (E) susceptibility results. The figure was modified from a screenshot captured in October 2021. The updated and complete contents of these tables can be found on the web (https://covdb.stanford.edu/search-drdb/?host=human&mutations=S%3A484K).

**Fig 4:**
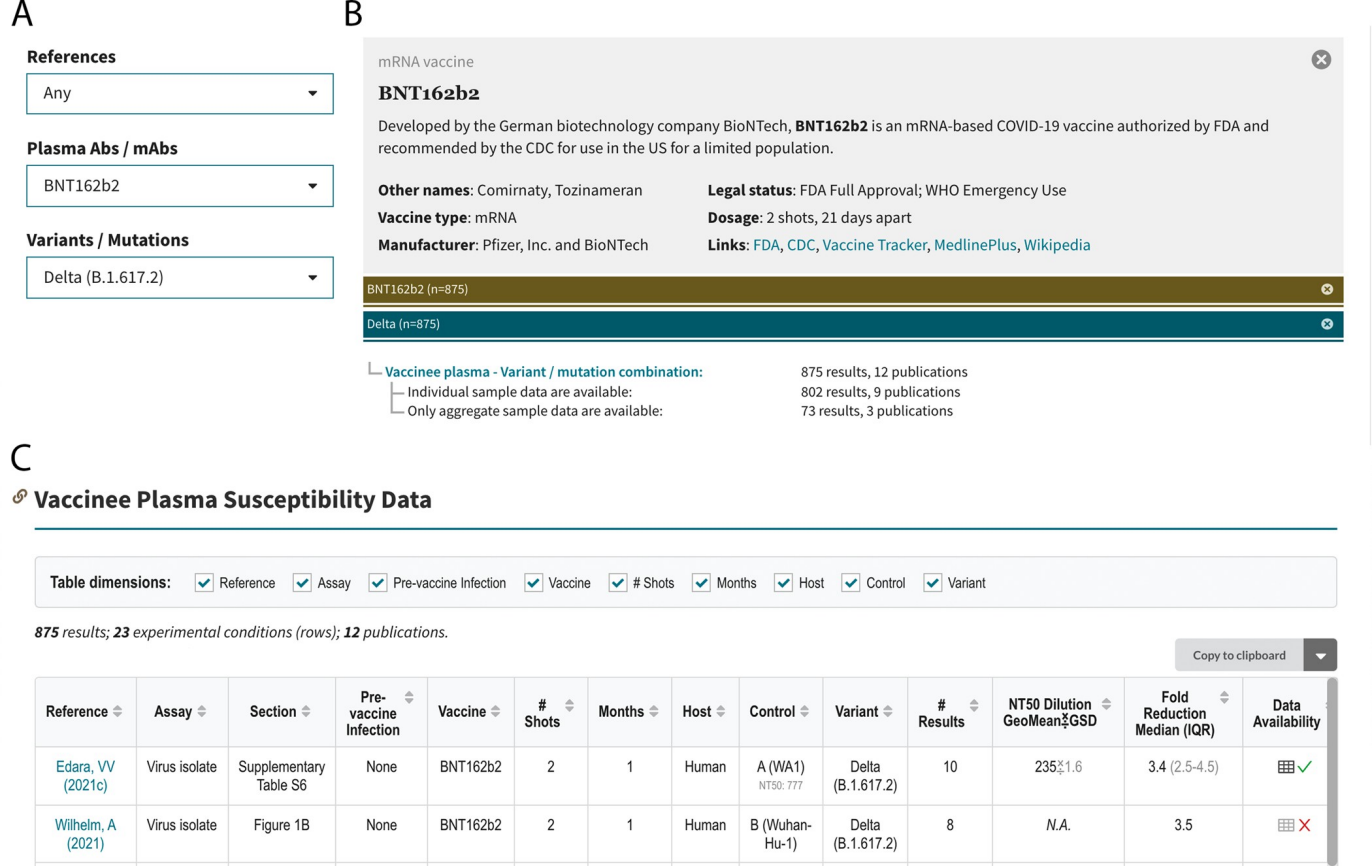
Query interface, sample query, and outline of query results for an example query for the susceptibility of the SARS-CoV-2 Delta variant to the vaccinee plasma (VP) of persons who received the BNT162b2 vaccine. The query interface containing three dropdown boxes is shown at the upper left (A). BNT162b2 is selected from the “Plasma / mAbs” dropdown box, and the Delta variant is selected from the “Variants / Mutations” dropdown box. The upper right summarizes the data returned by the query, which in this case includes 875 results from 12 publications (B). The summary distinguishes between results for which only aggregate (mean or median) data are provided and results for which individual data are provided. The section below the header shows the header and first few rows of the table entitled “Vaccinee Plasma Susceptibility Data” (C). The figure was modified from a screenshot captured in October 2021. The updated and complete contents of this table can be found on the web (https://covdb.stanford.edu/search-drdb/?host=human&vaccine=BNT162b2&variant=Delta).

The query interface allows users to search the database using one or more of the following three criteria: (i) published reference; (ii) antibody preparation (mAb, CP, or VP); and (iii) SARS-CoV-2 variant or spike mutation(s). If the “References” dropdown option is selected, then all the data associated with that reference is displayed in separate tables containing neutralization susceptibility data for mAbs, CP, and VP and/or *in vitro* selection data for mAbs. If the “Plasma / mAbs” dropdown option is selected, users must select either a specific mAb, CP from a person infected with a specific variant, or VP from persons who received a specific vaccine. If the “Variants / Mutations” dropdown option is selected, users must select a particular VOC, VOI, other variant, individual spike mutation, or combination of spike mutations. Selecting items from multiple dropdown boxes restricts the output to the data specified by the combination of dropdown items. Fig 3 shows the output returned by selecting E484K from the “Variants / Mutations” dropdown. Fig 4 shows the output returned by selecting BNT162b from the “Plasma / mAbs” dropdown and the Delta variant from the “Variant / Mutations” dropdown.

#### Query results

The mAb, CP, and VP susceptibility query result tables contain between 10 and 13 column headers (Fig 3C–3E). The mAb table contains column headers indicating the reference and location of the data within each reference (e.g., figure or table); assay type (e.g., pseudotyped virus); mAb tested; variant tested; IC_50_ in ng/ml; and fold-reduced susceptibility compared with a control virus (that is also present in the table; Fig 3C).

The VP table contains column headers that indicate the reference and location of the data within the reference, type of assay used, vaccine received, number of immunizations, number of months since immunization, whether the sample was obtained from a vaccinated person with confirmed prior infection, variant tested, geometric mean neutralizing titer, and median fold reduction in titer compared with the control virus (Figs 3D and 4C).

The CP table contains column headers that indicate the reference and location of the data within the reference, assay type, lineage of the virus that infected the person from whom the CP was obtained, number of months between infection and the plasma sample, variant tested, geometric mean neutralizing titer; and median fold reduction in titer compared with the control virus (Fig 3E).

Each table also contains two additional columns: “# Results” and “Data Availability”. The number of results indicates the number of neutralizing experiments. The data availability contains a “√” if individual measurements are available or an “X” if data are only available in aggregate (i.e., as a geometric mean titer or a median fold reduction in susceptibility). If the individual measurements are available, they can be viewed by clicking on the spreadsheet icon in the “Data Availability” column. This will create a pop-up table containing the data, which can then be copied to the user’s clipboard. The “Download CSV” tab at the top of each table allows users to download all rows for analysis.

#### Interacting with the query results

**Fig 5:**
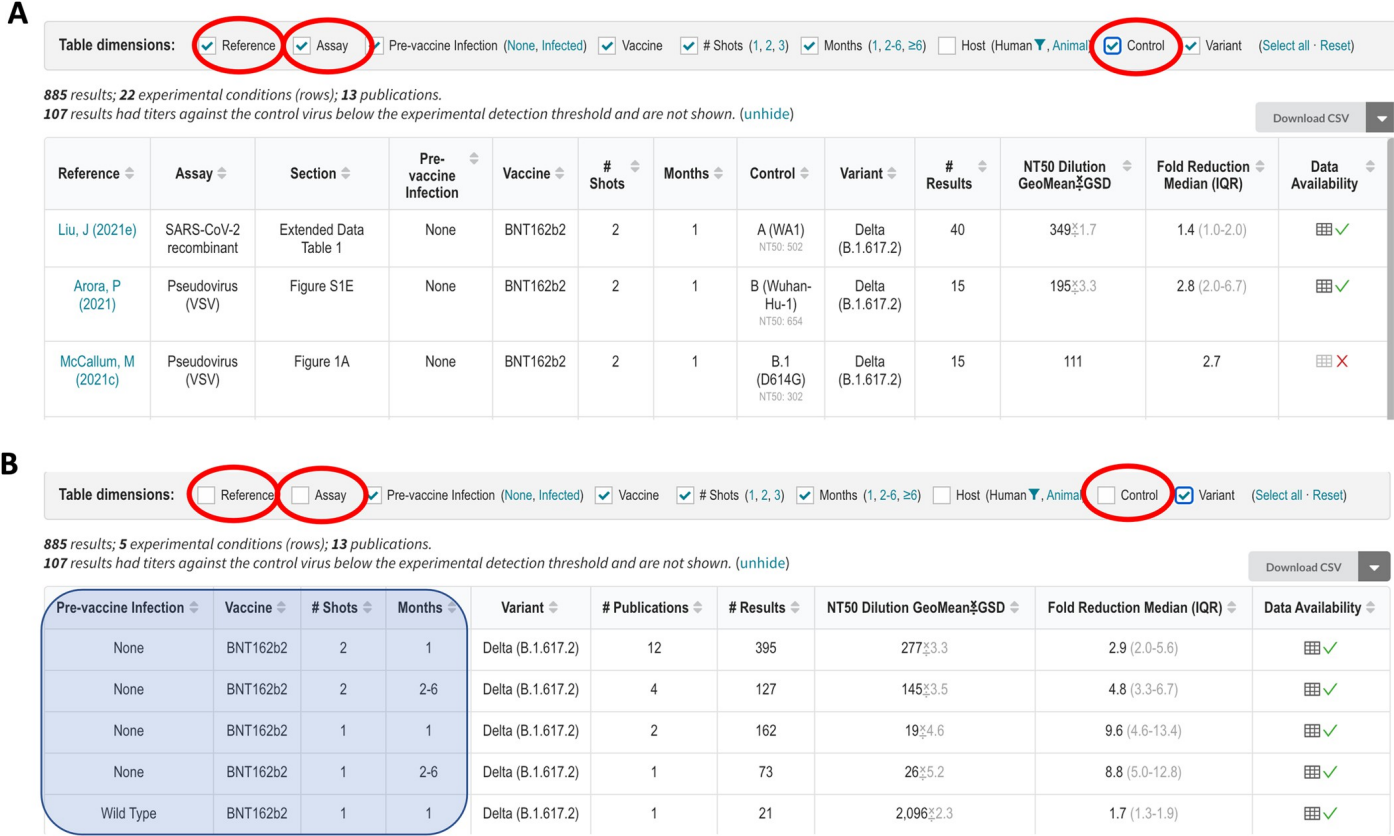
Aggregating the query results for the example shown in Fig 4. The top part of the figure shows the first three of 22 experimental conditions defined by the column headers in which BNT162b2-associated VP was tested for activity against the Delta variant (A). The results for each experimental condition are shown in different rows each containing the geometric mean of the neutralizing antibody titer and the median fold-reduction in titer compared with a control virus. The bottom part of the figure shows that the 22 rows can be displayed using 5 rows by aggregating those results obtained from different references using different assays or different control viruses by deselecting the “Reference”, “Assay”, and “Control” variant (checkboxes within red ovals). The neutralization data are now aggregated according to the number of BNT162b2 immunizations, time since immunization, and whether the VP was obtained from a person who had been infected prior to vaccination (shaded area superimposed on the first four columns). The number of experimental results in each row in Fig 5B is increased because each row may now contain data from more than one reference.

Each table can be considered to have multiple dimensions represented by the table’s column headers. For example, Fig 5A indicates the table dimensions for the query output with BNT162b2 selected from the “Plasma Abs / mAb” dropdown and the Delta variant selected from the “Variants / Mutations” dropdown. The table contains 22 rows (i.e., experimental conditions) summarizing 885 results obtained from 13 references. The table dimension check boxes enable users to aggregate the data in the table by deselecting one or more dimensions. For example, if the user deselects the “Reference”, “Assay”, and “Control” variant dimensions, the data are summarized with six rows that provide the median fold-reduction in susceptibility to BNT162b2-associated VP for the Delta variant according to the number of vaccinations received, the time since vaccination, and whether the VP was obtained from a previously vaccinated person (Fig 5B).

### Sequence analysis program

The CoV-RDB website contains a SARS-CoV-2 sequence analysis program that leverages the code base written for the widely used Stanford HIV Drug Resistance Database interpretation program [20, 21]. The CoV-RDB sequence analysis program supports three types of input: (i) a list of spike mutations; (ii) one or more consensus FASTA sequences containing any part of the SARS-CoV-2 genome; and (iii) one or more codon frequency table (CodFreq) files containing the following seven columns: gene, amino acid position, number of reads at that position, codon, amino acid encoded by the codon, number of reads for that codon, and proportion of reads for that codon. An auxiliary program provided through the website or via download enables users to convert next generation sequencing (NGS) FASTQ files into human and machine-readable CodFreq files that are much faster to analyze. CodFreq files make it possible to estimate the extent of background noise resulting from sequencing or experimental artifacts (S2 Fig) [21].

If one or more spike mutations is submitted, the CoV-RDB sequence analysis program reports information on specific mutations and generates summary tables containing the susceptibility of viruses with these mutations to mAbs, CP, and VP (S3 Fig). If a FASTA sequence is submitted, the program returns comments, summary tables, a list of the SARS-CoV-2 genes in the submitted sequence, a list of each of the amino acid mutations in each virus gene, and the sequence’s PANGO lineage assignment (S3 Fig). If a CodFreq file is submitted, the program returns all the above information and summarizes the read coverage for each position along the genome and provides users with options to select read depth and mutation-detection thresholds below which mutations will not be reported. If multiple sequences are submitted, users have the option of obtaining all the results in a single downloadable CSV file.

**Fig 6:**
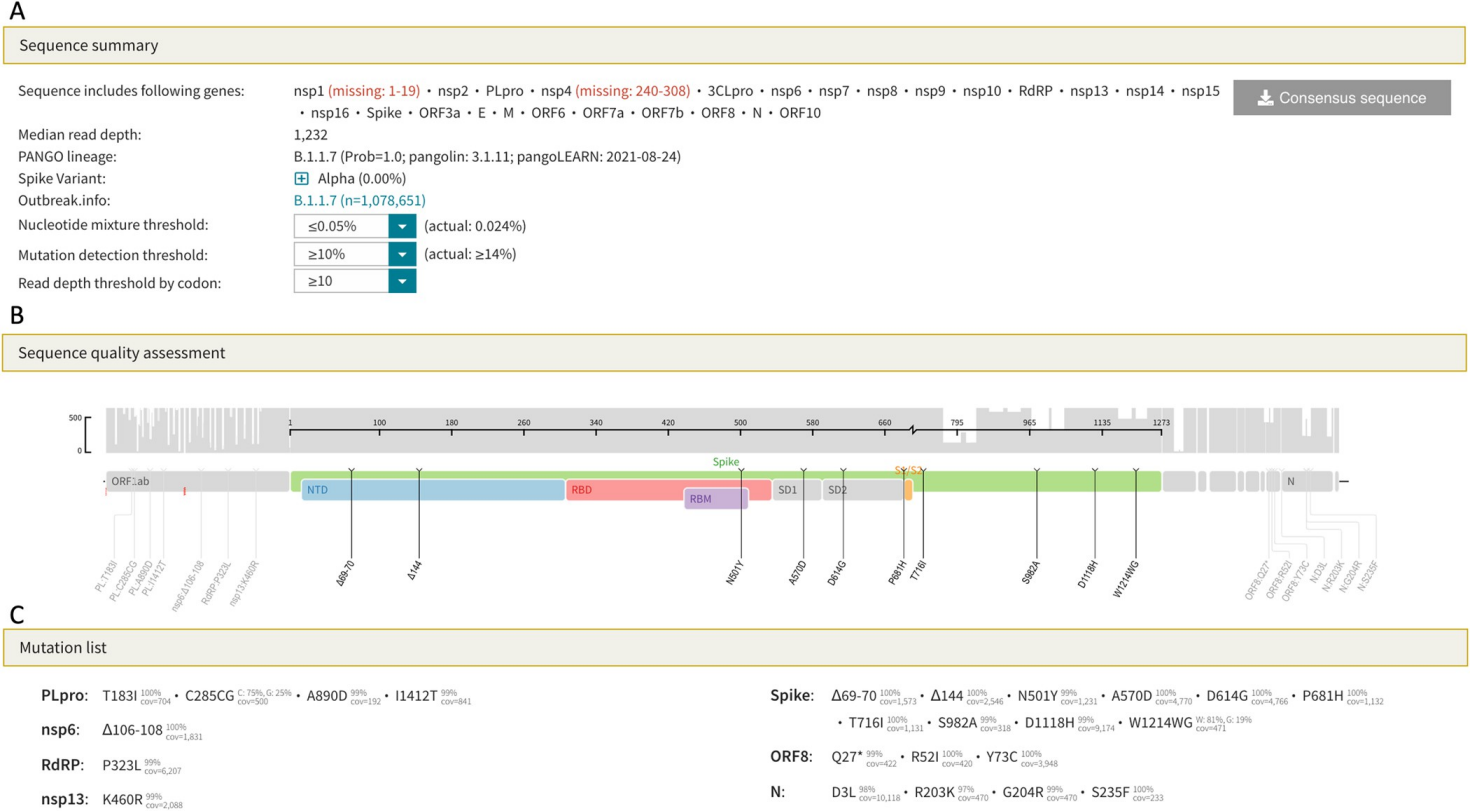
SARS-CoV-2 sequence analysis program output for FASTQ sequences or codon frequency tables. The sequence summary section (A) lists the genes that were sequenced, the median read depth, and the PANGO lineage. This section also contains dropdown boxes that enable the user to select the minimum number and proportion of reads, respectively, required to identify a mutation. The threshold that minimizes the proportion of positions with nucleotide ambiguities can also be selected. The sequence quality assessment section displays the read depth across the genome and lists only those amino acid mutations that meet the user-defined criteria specified in the sequence summary section (B). The mutation list section lists those amino acid mutations that meet the user-defined criteria and shows the proportion of reads containing the mutation (C). The output shown in this figure can be regenerated by loading the example file B.1.1.7 (ERR5026962) at this URL: https://covdb.stanford.edu/sierra/sars2/by-reads/.

Fig 6A–6C show three parts of the output generated when either a FASTQ sequence or CodFreq file is submitted to the sequence analysis program: sequence summary (Fig 6A), sequence quality assessment (Fig 6B), and mutation list (Fig 6C). The mutation comments and neutralization susceptibility data, which are also included in the output, are not shown. The sequence summary section (Fig 6A) lists the genes that underwent sequencing, the median read depth, and the PANGO lineage. This section also contains three dropdown boxes that help users select the appropriate threshold for identifying sub-consensus mutations. The read depth and mutation detection thresholds are used to select the minimum number and proportion of reads required for a mutation to be considered viral in origin rather than an experimental artifact. The nucleotide mixture threshold allows users to select a threshold which minimizes the number of nucleotide ambiguities present in the sequence.

## Discussion

Because SARS-CoV-2 is a rapidly evolving RNA virus infecting millions of people worldwide, new variants will continue to emerge and influence regional and global pandemic trajectories. Ongoing epidemiological and clinical studies are needed to understand the risk of re-infection and vaccine failure posed by different variants. However, because the spectrum of SARS-CoV-2 variants is expanding and shifting faster than epidemiological studies can be conducted, laboratory-based markers will increasingly be used to identify those variants that could predispose to re-infection and vaccine failure. SARS-CoV-2 neutralizing antibody titers are correlates of immune protection and are likely to be useful as an endpoint in vaccine trials and to determine the need for vaccine boosters and immunogen updates.

CoV-RDB is the only database to comprehensively curate published data on the neutralizing susceptibility of SARS-CoV-2 variants and spike mutations to mAbs, CP, and VP. Although non-neutralizing Abs and cellular immune responses also contribute to protection from infection, the presence of neutralizing antibodies targeting the spike protein has correlated most strongly with protection from infection in animal models and in previously infected and vaccinated persons [22–25], Neutralizing susceptibility assays are also better suited to standardization compared with assays of other immunological defense mechanisms.

CoV-RDB can be downloaded in its entirety without restrictions. This is accomplished using a dual database pipeline that combines the full-fledged PostgreSQL database system to enforce relational data integrity and the simplicity of the SQLite database system to enable users to download and query the database without the overhead of accessing a host server. By making the database fully available to all users, we aim to encourage data sharing and the editing of the underlying CSV files by the authors of published studies.

CoV-RDB neutralizing susceptibility query output can be considered multidimensional tables in which the rightmost columns contain numerical results (e.g., titers and fold-reductions in susceptibility) while the leftmost columns contain experimental conditions. The experimental conditions are explanatory variables that either directly influence neutralizing susceptibility (e.g., specific vaccine, time since vaccination, and SARS-CoV-2 variant) or have a more subtle effect on susceptibility (e.g., type of neutralizing assay and control virus). The CoV-RDB query interface enables users to explore the query results at different levels of granularity by filtering or aggregating them according to one or more experimental conditions without making additional calls to the web server.

The sequence analysis program shares many features with the Sierra HIV Drug Resistance Database sequence analysis program. For the analysis of NGS data, both programs use Minimap2 [26] to align individual reads to a reference sequence resulting in SAM/BAM files and SAM2CodFreq, a program we wrote to create CodFreq files. The CodFreq format has several advantages over the commonly used variant call format (VCF) because it can be interpreted without a reference sequence and can be used independently from the accompanying SAM file. CodFreq files have a simple tabular format enabling them to be viewed and manipulated using a spreadsheet. The sequence analysis program uses the CodFreq files to assist users determine the appropriate threshold for distinguishing background sequence artifact from authentic sub-consensus amino acids.

### Study limitations

Neutralizing susceptibility data are highly heterogeneous occasionally resulting in discordant results across studies [14, 17, 27]. There are three main sources for this heterogeneity. First, the composition of neutralizing antibodies among previously infected and vaccinated individuals is heterogeneous [6, 28]. Second, there are different types of neutralizing assays including those performed in cell culture using pseudotyped viruses, chimeric viruses, recombinant viruses, and clinical isolates and, more recently, surrogate neutralizing assays that assess the ability of antibodies to block the interaction between the SARS-CoV-2 RBD and ACE2. Third, results for the same sample against a virus variant can differ even among laboratories using the same type of assay as a result of differences in virus inoculum size, the cells used for culture, and viral replication endpoints [14, 17, 27]. As neutralizing assays become more standardized and as external controls such as those provided by the WHO [29] are increasingly used, it is likely that reproducibility across studies will improve.

Data on the clinical significance of SARS-CoV-2 neutralizing susceptibility is continually evolving [22, 23, 30–34]. The utility of neutralizing antibodies as a correlate of immune protection is ultimately determined in epidemiological studies. Therefore, a database devoted to protective immunity should ideally contain both laboratory and epidemiological data. The main obstacle to expanding CoV-RDB to also include epidemiological studies of vaccine efficacy is that such studies are much more complex than those reporting *in vitro* neutralizing data. For example, vaccine efficacy data depends not just on the vaccine, the variant, and the time since vaccination but also on the study design and the age and immune status of the study population. Moreover, in many vaccine-efficacy studies, the proportion of individuals infected with different variants is not known.

### Future directions

Although the CoV-RDB is centered around just four main entities (references, viruses, antibodies, and experiments), differences among the types of viruses, types of antibody preparations, and types of experiments has necessitated a sophisticated database design. Nonetheless, as new types of experiments are being published, database schema will require continued updating. For example, the use of an international external standard for calibration such as the one developed by the WHO will increase concordance across different assays and will be reported using international units rather than as an IC_50_ (for mAbs) or a plasma dilution [29, 35].

We have also added the comprehensive deep mutational scanning data published by the Bloom laboratory to the database [36–41]. The data from these studies are displayed only for those mAbs which have received FDA EUAs or are in advanced clinical development, and those mutations that have been reported to occur at a frequency above 0.001%. In addition to reporting the escape fraction associated with a mutation to an mAb, we report the level of protein expression within yeast of RBDs containing the mutation (a measure of protein stability) and the ACE2 binding of RBDs containing the mutation as mutations that bind poorly to ACE2 are less likely to be selected *in vivo*. Although binding data have been reported for many other mAbs using enzyme linked immunoassays, surface plasmon resonance, and biolayer interferometry, we have not curated these data as they have not been as comprehensive as the deep mutational scanning data.

Commercial total binding assays do not differentiate between binding and neutralizing antibodies. They also do not measure binding or neutralization of multiple variants but rather assess binding to pre-variant spike proteins. However, total binding assays often display moderately strong correlations with neutralizing assays [35, 42, 43] and activity against specific variants may eventually be assessed using variant-specific reagents. We may eventually add such data to CoV-RDB if they will provide insights that cannot be obtained solely from neutralizing antibody studies.

Although CoV-RDB contains neutralizing susceptibility data obtained using the plasma from infected animal experiments (e.g., non-human primates, hamsters, and mice), we have not included data from animal model challenge studies as such studies would require extensive modifications to our current database schema. Therefore, we will continue to monitor these studies and consider adding top-line data from these studies to alert database users to the existence of these studies without rigorously representing study details. Finally, a similar approach will be considered for studies of vaccine efficacy. We are therefore exploring the possibility of adding the top-line data of these studies so that the findings of these studies can be correlated with the *in vitro* neutralizing data in CoV-RDB.

## Supporting information

S1 Table Slightly different patterns of spike mutations within each of the SARS-CoV-2 Variants of Concern (VOCs) and Variants of Interest (VOIs). (XLSX)

S1 Fig Data management workflow for CoV-RDB. Weekly incremental searches of PubMed and preprint servers (BioRxiv/MedRxiv and Research Square) are performed. Publications that appear to have data pertinent to SARS-CoV-2 variants and their susceptibility to mAbs, convalescent plasma (CP), and vaccinee plasma (VP) are downloaded to a Zotero reference database folder to enable full-text review and data curation. Extracted data are exported into a set of linked CSV files in an open-source GitHub repository (https://github.com/hivdb/covid-drdb-payload). Extracted data are then imported into a PostgreSQL database where the data are validated for completeness and consistency before being exported as a single SQLite database file that serves as the back end for the CoV-RDB website and is available to users for download.
(TIF)

S2 Fig Creating a codon frequency file from a FASTQ file. FASTQ files are aligned to the consensus Wuhan-Hu-1 reference sequence using the Minimap2 alignment program. The resulting BAM/SAM files are then processed by a library SAM2CodFreq that we wrote to generate a codon frequency (CodFreq) file containing seven columns as shown on the right. The table here shows the results from three codons (spike positions 500 to 502). The observation that many codons shown in this (and other parts of the same file which are not shown) are present at levels between 0.2% and about 2% suggests that codons present at these low proportions likely represent sequencing or experimental artifacts (i.e., “background noise”). However, as the mutation N501Y occurs at a considerably higher proportion (34.3%), it is likely to be present in the infecting virus population.
(TIF)

S3 Fig Functions of the SARS-CoV-2 sequence analysis program. The program supports three types of input: a list of spike mutations; one or more consensus FASTA sequences containing any part of the SARS-CoV-2 genome; and one or more FASTQ sequences. However, because a FASTQ sequence can take several minutes to analyze, users are advised to first convert them to a codon frequency (CodFreq) file through an auxiliary program. If a list of spike mutations is submitted, the program returns comments about notable mutations and summary tables reporting the susceptibility of viruses with these mutations to mAbs, CP, and VP. If a FASTA sequence is submitted, the program returns the preceding information plus a list of the SARS-CoV-2 genes, the amino acid mutations in the sequence, and the sequence’s PANGO lineage. If a FASTQ sequence or codon frequency table is submitted, the program provides the preceding information and the read coverage for each position along the genome. It also provides users with the options to select read depth and mutation-detection thresholds below which mutations will not be reported.
(TIF)
