# Supplementary figures and images for: Coronavirus Resistance Database (CoV-RDB): SARS-CoV-2 susceptibility to monoclonal antibodies, convalescent plasma, and plasma from vaccinated persons
Source: PLoS One. 2022 Mar 9;17(3):e0261045. doi: 10.1371/journal.pone.0261045 (PMC8906623; doi:10.1371/journal.pone.0261045)

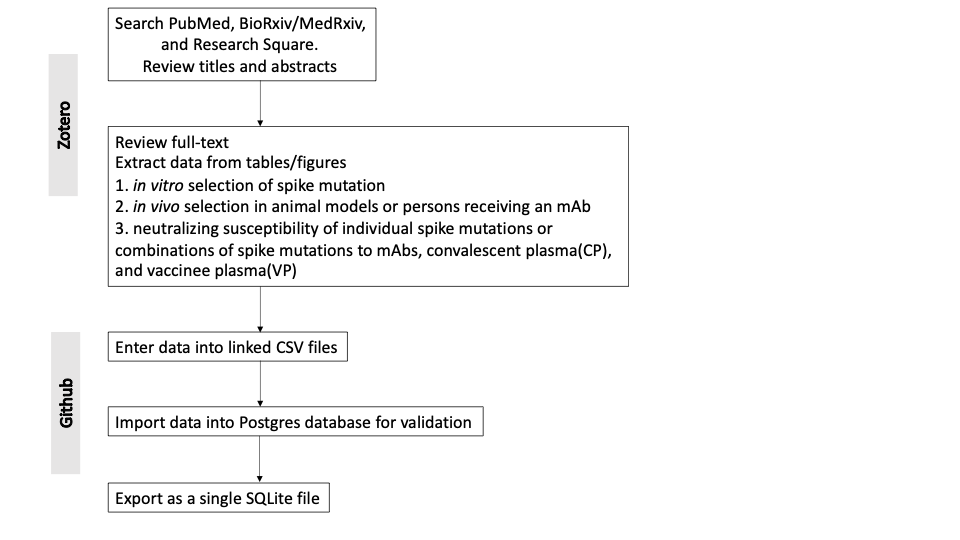

Supplement: S1 Fig — Weekly incremental searches of PubMed and preprint servers (BioRxiv/MedRxiv and Research Square) are performed. Publications that appear to have data pertinent to SARS-CoV-2 variants and their susceptibility to mAbs, convalescent plasma (CP), and vaccinee plasma (VP) are downloaded to a Zotero reference database folder to enable full-text review and data curation. Extracted data are exported into a set of linked CSV files in an open-source GitHub repository (https://github.com/hivdb/covid-drdb-payload). Extracted data are then imported into a PostgreSQL database where the data are validated for completeness and consistency before being exported as a single SQLite database file that serves as the back end for the CoV-RDB website and is available to users for download. (TIF) [file pone.0261045.s002.tif]

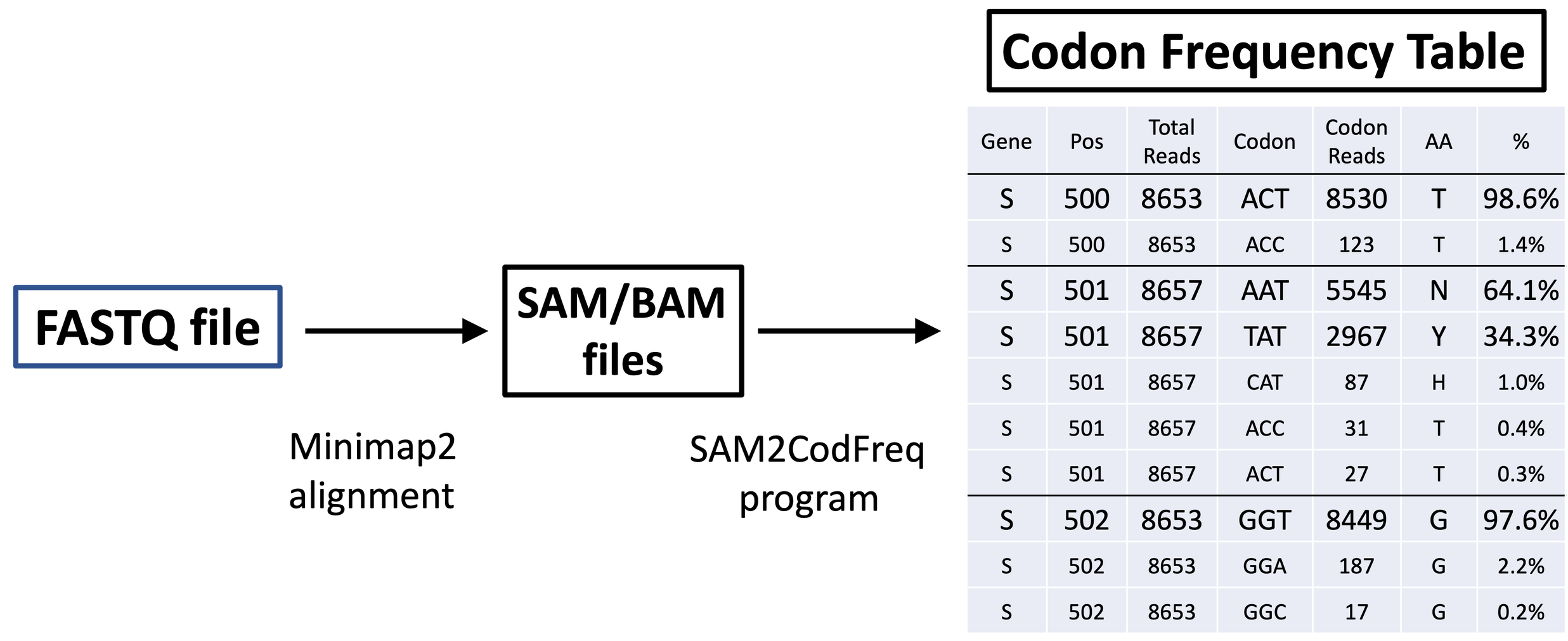

Supplement: S2 Fig — FASTQ files are aligned to the consensus Wuhan-Hu-1 reference sequence using the Minimap2 alignment program. The resulting BAM/SAM files are then processed by a library SAM2CodFreq that we wrote to generate a codon frequency (CodFreq) file containing seven columns as shown on the right. The table here shows the results from three codons (spike positions 500 to 502). The observation that many codons shown in this (and other parts of the same file which are not shown) are present at levels between 0.2% and about 2% suggests that codons present at these low proportions likely represent sequencing or experimental artifacts (i.e., “background noise”). However, as the mutation N501Y occurs at a considerably higher proportion (34.3%), it is likely to be present in the infecting virus population. (TIF) [file pone.0261045.s003.tif]

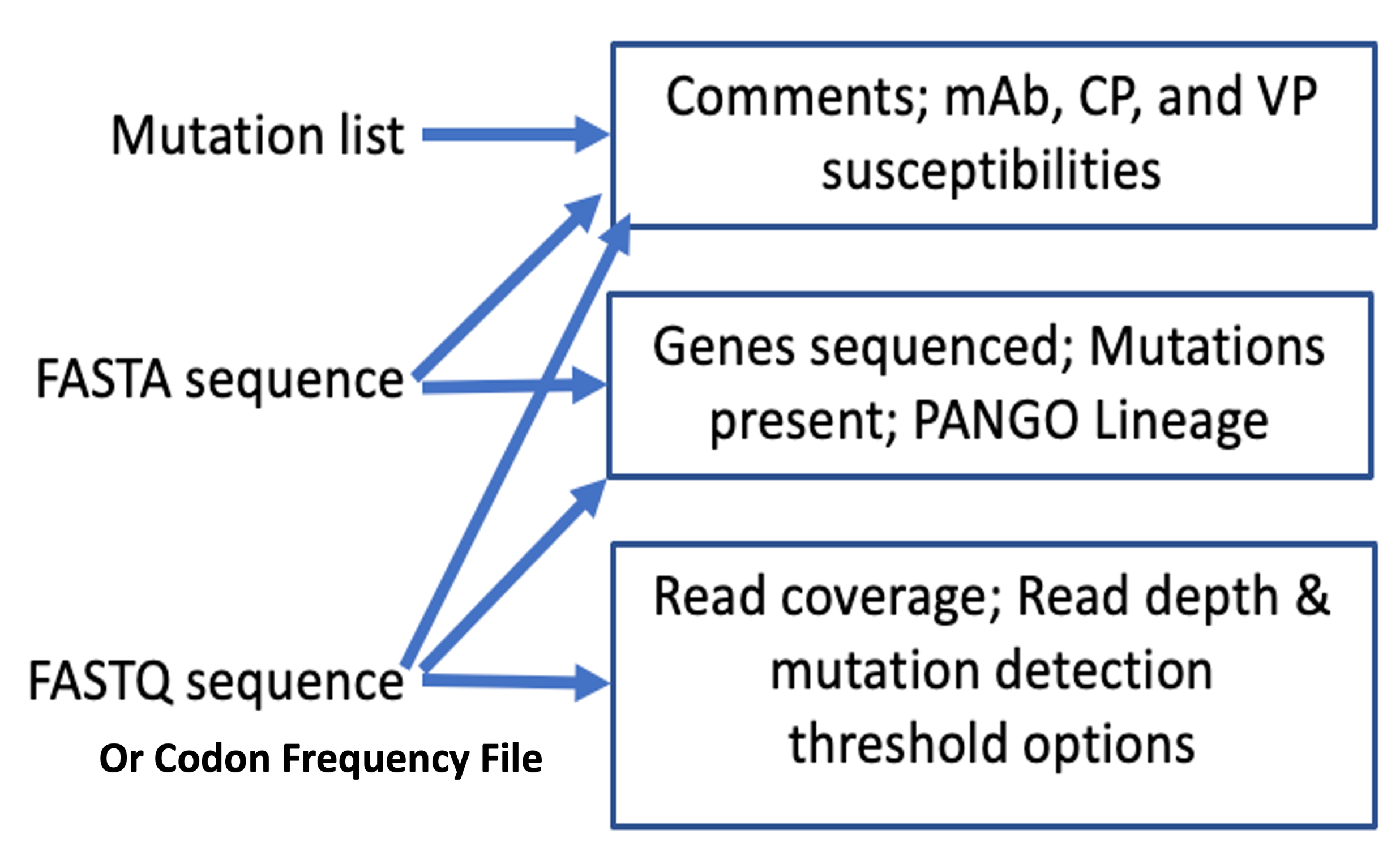

Supplement: S3 Fig — The program supports three types of input: a list of spike mutations; one or more consensus FASTA sequences containing any part of the SARS-CoV-2 genome; and one or more FASTQ sequences. However, because a FASTQ sequence can take several minutes to analyze, users are advised to first convert them to a codon frequency (CodFreq) file through an auxiliary program. If a list of spike mutations is submitted, the program returns comments about notable mutations and summary tables reporting the susceptibility of viruses with these mutations to mAbs, CP, and VP. If a FASTA sequence is submitted, the program returns the preceding information plus a list of the SARS-CoV-2 genes, the amino acid mutations in the sequence, and the sequence’s PANGO lineage. If a FASTQ sequence or codon frequency table is submitted, the program provides the preceding information and the read coverage for each position along the genome. It also provides users with the options to select read depth and mutation-detection thresholds below which mutations will not be reported. (TIF) [file pone.0261045.s004.tif]
